# Environmental and occupational respiratory diseases – 1063. Spirometric abnormalities in non smoking bus drivers of hyderabad

**DOI:** 10.1186/1939-4551-6-S1-P61

**Published:** 2013-04-23

**Authors:** Mohammed Faisal, Prasad Eswara Chelluri, Saikrishna Singaraju, Jayasri Helen Gali, Sayeed Ahmed, P Srinivas

**Affiliations:** 1 Pulmonology, Shadan Institute of Medical Sciences, Hyderabad, India

## Aim

The prevalence of COPD in non-smoking bus drivers of Hyderabad city (A.P State Road Transport Corporation).

## Background

Hyderabad metro has high atmospheric pollution with a mean TSPM 280 mg/cubic meter of air, sometimes as high as 400.

Vehicles contribute 50% of the total air pollution in urban areas [1].

## Subjects

82 Non-smoking male bus drivers of APSRTC. Buses are non-air-conditioned with open windows.

## Methods

An occupational-demographic data was obtained and American Thoracic Society – Division of Lung Disease (ATS) Questionnaire administered. Subjects were enrolled between May and September 2012. Spirometry performed using an auto calibrated system (Microlab, Kent, UK) and abnormalities were classified and graded (GOLD Guidelines 2005). The data were statistically treated for correlation between symptoms, spirometric abnormalities and work experience.

## Results

Age 38±8.75yrs; BMI 23.9±2.8

COPD was diagnosed in 35 out of 82 (42.6%) based on spirometry. COPD was “mild” in 16 (45%), “moderate” in 18 (51.4%) and “severe” in 1 (2.8%). 13 of 30 subjects with work experience of >10 years had COPD. 22 of 52 subjects with work experience of <10 years had COPD (Fishers exact test (p<0.01).

The spirometric indices demonstrated difference between Group A i.e. drivers with >10years experience and Group B <10 years. FEV 1 was lower in Group A vs. Group B (2.49± 0.65 Vs 2.73± 0.49; p =0.06). PEFR (5.42± 2.11 Vs 6.48±1.91; p=0.02), FEV6, FEV1/FEV6, FEF2575, post bronchodilator reversibility were lower in Group A vs. Group B though statistically not significant.

Symptom profile: shortness of breath in 35 (cough in 12 (14.6%), morning sputum in 15 (18%), wheeze in 6, recurrent sinusitis in 7 (8%).

## Conclusion

The present sample study of non smoking bus drivers in an urban area of India using ATS questionnaire and spirometry is, perhaps, the first to the best of our knowledge. About 40% of the subjects had COPD. Environmental preventive measures required.
